# Macrodactyly

**DOI:** 10.3390/children11070753

**Published:** 2024-06-21

**Authors:** Kaja Giżewska-Kacprzak, Maximilian Śliwiński, Karol Nicieja, Lidia Babiak-Choroszczak, Ireneusz Walaszek

**Affiliations:** 1 Department of Pediatric and Oncological Surgery, Urology and Hand Surgery, Pomeranian Medical University in Szczecin, 1 Unii Lubelskiej Street, 71-252 Szczecin, Poland; maximilian.sliwinski@pum.edu.pl (M.Ś.);; 2 Department of Nursing, Faculty of Health Sciences, Pomeranian Medical University in Szczecin, 48 Żołnierska St., 71-210 Szczecin, Poland

**Keywords:** macrodactyly, overgrowth, congenital upper limb difference, PROS, hand surgery

## Abstract

Macrodactyly is a rare congenital limb difference manifesting as an overgrowth of one or more fingers or toes. The pathological process affects all tissues of the ray in the hand or foot. The enlargement can significantly alter the limb’s appearance and impair its function. The role of a pediatrician is to distinguish isolated macrodactyly from syndromic conditions (including PIK3CA-Related Overgrowth Spectrum) or mimicking conditions to enable early interdisciplinary consultation and treatment planning. The psychological stigma associated with this often disfiguring condition necessitates support for patients and their family. We present a practical guide for physicians who might be the first to raise suspicion of macrodactyly and initiate further diagnostics to achieve adequate treatment and support for children and caregivers.

## 1. Introduction

Macrodactyly, originating from the Greek word “makros”, meaning large, and “daktylos”, meaning digit, is a rare congenital difference of the hand and/or foot presenting as an overgrowth of one or more rays. The enlargement can affect all tissues, including bones, muscles, adipose, and nerves. It may impair limb function and inflict significant psychological stress on both children and their families [1,2]. It can be an isolated disorder or part of a syndrome [1,2]. In recent years, significant progress in understanding its pathophysiology has led to improved diagnostic and treatment strategies, offering hope for better outcomes. Therefore, pediatricians who may see children affected by macrodactyly as the first physicians should have a basic understanding of the condition. We collected up-to-date information as a practical guide for pediatricians to raise awareness about macrodactyly as a rare entity that requires an interdisciplinary approach and a suitable treatment protocol for patients and their parents.

## 2. Clinical Picture and Classification

**Figure 1:**
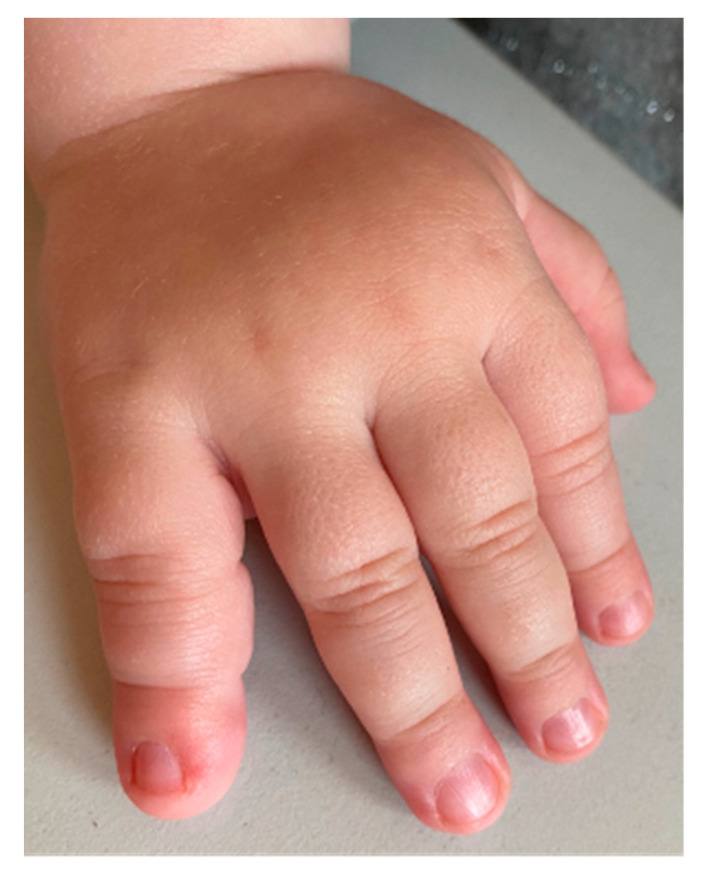
Isolated macrodactyly of the little finger of the hand.

**Figure 2:**
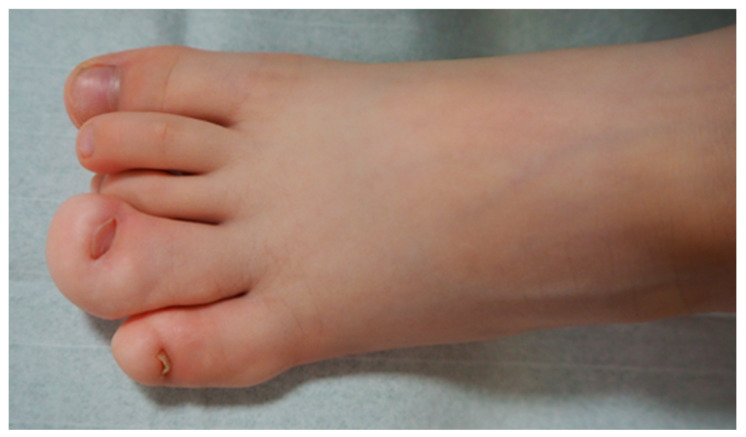
Foot macrodactyly of the 4th and 5th rays.

Macrodactyly is characterized by an increase in the size of one or multiple digits in the hand or foot compared to the contralateral or neighboring rays [3]. Excessive growth can occur bilaterally. The affected fingers frequently show asymmetrical deformity, stiffness, limited mobility, discomfort, or pain. Each patient may have a different proportion of overgrowth of bones compared to extensive fat tissue volume. In some children, overgrowth is accompanied by syndactyly, a congenital limb difference where fingers are joined together. Vascular anomalies, metacarpal involvement, or limb hemihypertrophy may also accompany it. Even with preserved function, visual asymmetry may lead to stigmatization and stress for the whole family [4,5] (Figure 1). Children with macrodactyly often struggle with everyday tasks or participation in childhood games. If a foot is affected, one of the first complaints can be a difference in shoe size or a need for custom-fitted shoes. It is worth noting that patients with foot macrodactyly tend to delay consultation despite its more progressive nature [1] (Figure 2). 

Each patient’s clinical presentation is unique, and regular controls are necessary to appreciate the growth rate over time through measurements at regular intervals. The condition’s severity may significantly vary, presenting either as static enlargement following the regular development rate or progressive overgrowth at a much faster pace than the unaffected limb. For more than 150 years, various authors have attempted to classify mysterious overgrowth deformities. Evolving theories about the condition have influenced the criteria for the suggested classifications [4]. Recent progress in comprehending the underlying somatic genetic factors causing overgrowth conditions calls into question past classifications. For practical reasons, e.g., communicating between professionals and parents, a simplified classification based on individual growth, associated conditions, and structure ratings (Table 1) holds clinical value [4].

Macrodactyly has its place in the internationally accepted OMT (Oberg–Manke–Tonkin) Classification for congenital upper limb anomalies. In the updated version of the OMT, isolated macrodactyly is classified in the dysplasias as a variant of the growth category, whereas syndromic cases are categorized in a separate group [6]. 

## 3. Epidemiology

The prevalence of macrodactyly is challenging to assess as the evidence is usually based on surgically treated patients. The data ranges from 1 in 200,000 (US) to 1 in 50,000 (Sweden), depending on the studied population. Some reports suggest that lower limbs are affected more frequently, from 1 in 50,000 to 1 in 18,000 [4]. There are limitations to the studies based on case series. However, looking into the future, nationwide registries such as the Congenital Upper Limb Differences Registry (CoULD) may provide better insight into children who do not undergo surgical intervention or who present late with the condition. The registry inclusion effect has been linked to an approximate one-third rise in the prevalence of analyzed conditions, with its dynamics stabilizing within the third year of reporting [7].

## 4. Pathophysiology and Genetics 

The etiology of macrodactyly is the subject of ongoing research. Historically, clinical presentations have provoked an explanation of pathophysiology with a simultaneous proposition of classification [4,8]. For example, repeatable cases of macrodactyly affecting multiple digits in the median nerve territory have led to a division of the condition into the following subtypes: type I with lipofibromatous hamartoma of a peripheral nerve, and type II with digital gigantism associated with neurofibromatosis and hyperostotic macrodactyly. A suggested theory positing digital nerve participation in the overgrowth raised hopes for nerve longitudinal resections to stop the growth. Unfortunately, the results were not satisfactory. Further discoveries brought to attention a different pathophysiological understanding [9].

The discovery of the role of the phosphatidylinositol-4,5-bisphosphate 3-kinase subunit alpha (PIK3CA) somatic gain-of-function mutations in the overgrowth led to the establishment of the PIK3CA-Related Overgrowth Spectrum (PROS) [10]. Since Rios et al. discovered PIK3CA mutations in tissue resected from patients with macrodactyly, the condition was also added to the PROS [9,11]. Hand surgeons had to understand that tissue genetic mosaicism underlines the continuous growth. The expectations of surgical treatment had to be adjusted with the understanding that a scalpel cannot change the infinite scheme of altered growth. The severity of mutations and their tissue specificity varies highly as they can occur at different times during embryogenesis, with macrodactyly developing later [12,13]. PIK3CA mutations activate a well-known oncogenic PI3K/AKT/mTOR (AKT, serine/threonine kinase; mTOR, a mechanistic target of rapamycin kinase) signaling pathway [14]. According to the literature, even though they are common in different types of cancer, PROS is not associated with an increased risk of malignancy [15]. Although there have been a few reported cases of neoplasms, there are still insufficient data to support associations [16]. It is worth pointing out that these mutations are somatic and, therefore, are not inherited [17]. Communicating this information to parents is crucial as they may feel guilty about their child’s condition [5].

## 5. Diagnostics

Clinical examination of the whole child is crucial to screen for complex conditions. Isolated macrodactyly should be distinguished from an overgrowth of the upper limb with continuous vascular malformation of the thorax. Isolated conditions have to be separated from syndromes. An X-ray of the affected limb usually supports asymmetry in the skeleton; however, in some cases, the soft tissue component can dominate over mildly overgrown bones. Classic radiograms can present joint deformities, osteophytes, and osteoarthritic changes in advanced cases [1]. Magnetic resonance imaging, computed tomography, and vascular studies may be required to assess more complex syndromic cases. The importance of consulting with other specialists cannot be overstated. It is mandatory to rule out a syndromic component of macrodactyly as external asymmetry may only be the tip of the iceberg of a condition, as observed in PROS. As mentioned, confirmation of PIK3CA mutations in biopsied tissue is of significant value. However, PIK3CA mutations are confirmed in only two-thirds of patients. This results from the low detection rate of macrodactyly as an isolated PROS compared to syndromic cases [18]. Therefore, in most cases, the diagnosis is based only on clinical manifestations [19]. The banking of material may benefit future studies as our understanding of overgrowth syndromes will hopefully improve. The prenatal diagnosis of macrodactyly has been reported [20] and theoretically can enhance postnatal vigilance towards syndromic presentations. 

## 6. Differential Diagnosis

**Figure 3:**
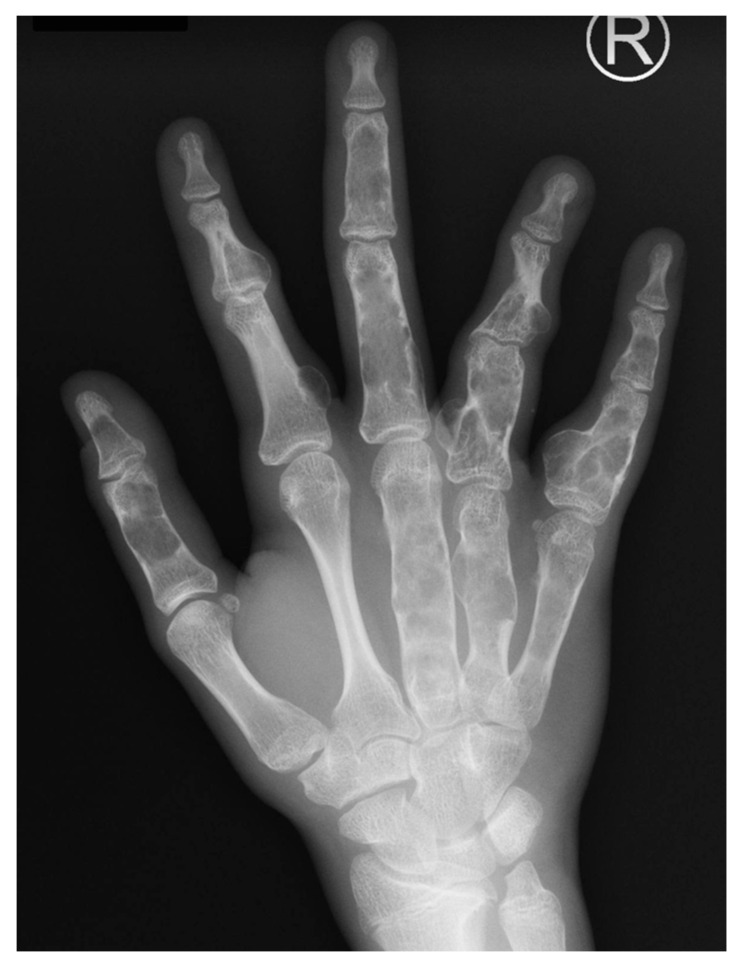
Multiple enchondromas of the hand in a 15-year-old-boy.

At first, the differential diagnosis must exclude tumorous origin of the asymmetry and enlargement of the digit (Table 2). A finger can increase in width, length, and circumference due to soft tissue, bony tumors, or vascular or lymphatic malformation. Although rare, both benign and malignant tumors of the hand can occur in the pediatric population [21]. Sarcomas, in particular, require intricate interdisciplinary oncological consultation, detailed radiological assessment, and magnetic resonance imaging to plan the appropriate course of action, such as surgical excision or incision biopsy. It is important to note that dangerous conditions, such as the abovementioned sarcomas or primary bone tumors, may mimic a benign enlargement of the ray, including macrodactyly, when the skin is not infiltrated. Bony tumors can present as a single lesion of the finger, but multiple enchondromas may also mimic overgrowth (Figure 3). In cases where multiple lesions are confirmed using X-ray imaging, the diagnosis should be differentiated between Ollier disease, which is usually unilateral, and Maffucci’s syndrome, which is predominantly bilateral [22]. Furthermore, in Maffucci syndrome, multiple enchondromas are accompanied by vascular overgrowths that become malignant in 8.5% of patients.

**Figure 4:**
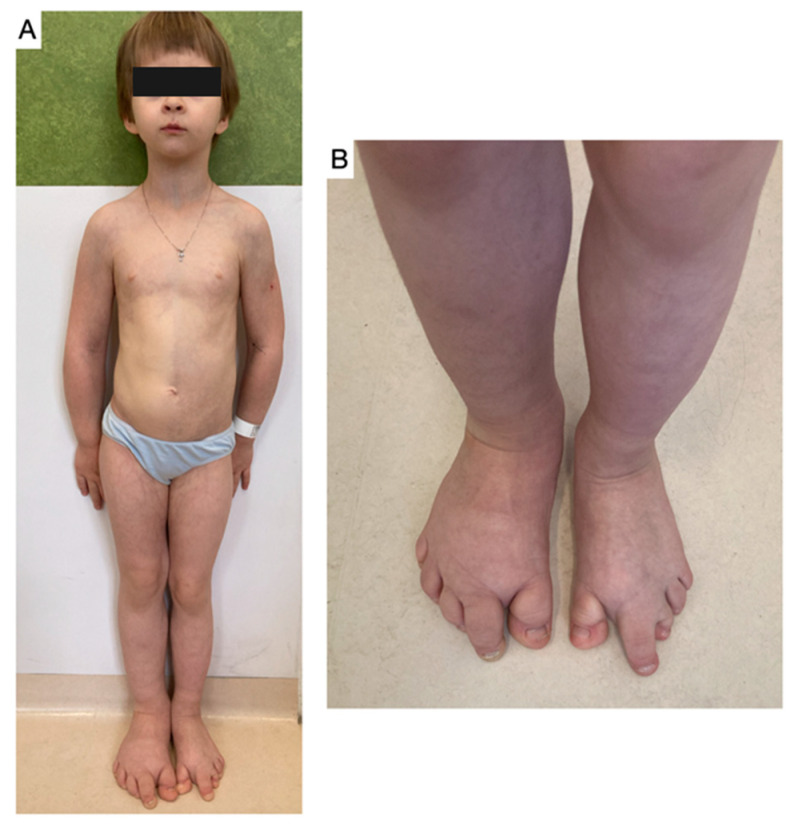
An 8-year-old girl with Klippel–Trenaunay Syndrome (**A**), with visible asymmetrical overgrowth of the bilateral II rays of both feet (**B**) as a component of the syndrome, along with hemihypertrophy and large vascular malformation of the thorax, abdomen, and right lower limb, accompanied by Vein of Galen malformation of the brain and right eye leucoma.

PROS is a wide range of syndromes that demand early suspicion and differential diagnosis for effective management (Table 3). Klippel–Trenaunay syndrome (Figure 4) exemplifies this with its complex vascular malformations and potential limb hypertrophy. The early suspicion of PROS should trigger an upscale in diagnostics to an interdisciplinary team, underlining the urgent need for each professional to play their part in confirming the diagnosis and enabling the early recognition of treatment options.

The National Institutes of Health Workshop Criteria include the following features in suspicion of PROS [23]:

1. Congenital or early childhood onset
2. **Two or more spectrum features**
  - Overgrowth: adipose, muscle, nerve, skeletal
  - Epidermal nevus
  - Vascular malformations: capillary, venous, arteriovenous, lymphatic
  **OR any one isolated feature**
  - Large, isolated lymphatic malformation
  - Truncal adipose overgrowth
  - Benign lichenoid keratoses
  - Seborrheic keratoses
  - HME (bilateral)/DMEG/focal cortical dysplasia type ll
  - Isolated macrodactyly or overgrown, splayed feet/hands, overgrown limbs
  - Epidermal nevus
3. Sporadic and mosaic overgrowth
4. Presence of a somatic PIK3CA mutation

## 7. Overgrowth Syndromes Associated with Neoplasms

An overgrowth may be the first sign of syndromes predisposing to malignancies [24]. Despite being rare, oncologists need to consult the patient and consider broader diagnostics. For instance, Beckwith–Wiedemann Syndrome (BWS) is associated with a higher risk of hepatoblastoma and Wilms tumor [25]. Clinical presentations vary and may include hemihypertrophy, macroglossia, facial dysmorphia, hypoglycemia, or abdominal wall defects [26]. Other overgrowth syndromes associated with pediatric neoplasms, such as Sotos syndrome or Simpson–Golabi–Behmel syndrome, are shown in Table 4. Enhanced oncological surveillance is crucial in patients who exhibit distinct features of these syndromes or have a positive family history [24,27]. In such cases, further oncological screening or genetic testing is recommended. A recent study found that even an isolated overgrowth can increase the risk of cancer by up to five times, highlighting the need for more research [25]. 

## 8. Interdisciplinary Approach

If the overgrown part of the limb is visible at birth, the neonatologist will be the first information provider for stressed parents. The main goal for pediatricians is to widen diagnostics to establish whether the condition is isolated or part of a syndromic presentation [5]. As mentioned, clinical examination with radiological findings is vital to exclude any tumorous causes of limb enlargement. Genetic counseling should be offered when data is collected. Surgical consultation with an experienced congenital upper limb professional may play an essential role in managing family and patient expectations. Overall surgical management focuses on enabling children to acquire manipulative skills and comfort in everyday activities with their friends while minimizing surgical intervention. However, in the context of PROS, the patient and caregiver journeys are complex matters. Rodrigues-Laguna et al. mapped the key milestones and experiences from birth to older adulthood using a patient-centered perspective [29]. Caregivers may face challenges caused by the stress of misdiagnosis or inaccessibility to genetic testing, whereas, later in life, patients may face pain or stigma. The need for assistance, inaccessibility in education, and social participation are the subject of psychological, psychiatric, social, and occupational therapy interventions that should be individually tailored. The role of support groups, which are usually self-organizing parent forums, cannot be overstated. They are a valuable, helpful, and informative resource that frequently fills the gaps in available information systems. Families share how they overcome their everyday struggles, doctor visits, and surgeries to offer hope, inspiration, and practical tips. Doctors should be aware of local or national initiatives in order to share their contact details with their patients [29]. 

## 9. Decision-Making in Macrodactyly

Offering appropriate treatment for macrodactyly is a challenging task, and the clinical presentation, the preferences of the patient’s family, and their default compliance potential should be considered. A thorough conversation with the parents is paramount for deciding on the most beneficial strategy. Connecting parents with children who have similar conditions and facilitating the exchange of their experiences can be beneficial. Psychological support should be considered as making a radical decision for a child at an early age is often the cause of stress for parents [1]. Parents’ quotes that children could decide about their limb once mature should be addressed with an educational presentation of the condition’s natural history and an up-to-date explanation of ongoing growth, avoiding commanding language. In static macrodactyly, where the disproportion in the size of the fingers is minimal and does not impair hand function, observation with regular follow-up should be proposed. Measurements and photographic documentation should be taken during each follow-up to collectively agree on further proceedings. In progressive presentations with severe overgrowth causing not only a cosmetic defect but also impairment of the function of the extremity, surgical treatment should be considered. Ezaki et al. summed up the decision-making process, emphasizing comparison the overgrown ray to the size of the parent’s finger size and the subjective stage of dysfunction and disfigurement [5]. This could help parents understand the benefits of early childhood interventions. 

## 10. Surgical Treatment

Due to potential persistent overgrowth, surgical interventions in macrodactyly are considered one of the most challenging in congenital hand surgery. The choice of surgical technique in isolated macrodactyly is based on numerous factors, such as which finger is affected, the number of overgrown rays, angulation, and the growth rate. The personal experience of surgeons is a significant part of the decision-making process. As the condition is rare, bias from individual experiences should be noted. The more that is understood about the genetic origins of ongoing growth, the more radical surgical options are considered. Acknowledging the risk of recurrence and repetitive operations promotes the consideration of amputations. Interventions have usually been divided into procedures for young, still-growing, and adult patients. Early interventions were supposed to modify and limit the growth, whereas later resections and arthrodesis focused on relieving pain and restoring lost function. 

**Figure 5:**
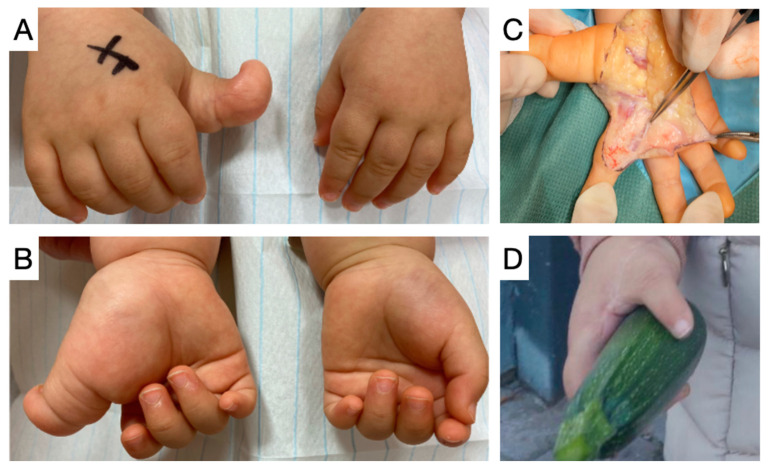
(**A**,**B**). Isolated macrodactyly of the thumb in a one-year-old female presenting with hyperextension in the interphalangeal joint, severely impairing function. (**C**). Thumb amputation with simultaneous pollicization of the index finger. (**D**). Long-term follow-up shows satisfactory functional and cosmetic effects of pollicization.

In cases where the finger of a child has already reached the size of an adult with angulation that affects function, surgical treatment is necessary. Multiple corrective osteotomies are procedures in which part of the bone is resected in a wedge shape to allow axial positioning of the remaining bone fragments. These fragments are then stabilized with removable implants, such as Kirschner wires. This technique can correct both lateral angulation and typical hyperextension of the interphalangeal joint. Simultaneous epiphysiodesis and destruction of the growth plates are usually performed. A significant volume of soft tissue can be debulked during surgical reduction of the fatty fibrotic part of the digit. Technically, digital nerves can be so enlarged and fibrotic that they mimic the surrounding fat tissue. Surgeons can longitudinally resect the nerves, but its vague effect on growth and the risk of loss of sensation make it questionable. However, there are case series where complete nerve resection in macrodactyly has been performed successfully, followed by allograft reconstruction. This is based on the hypothesis of the impact of hyperinnervation on the continued growth [30]. With an understanding of tissue mosaicism in the pathophysiology, parents should be informed about the high risk of recurrence of overgrowth and deformity. Sophisticated reconstructive techniques allow finger salvage with a decrease in digit size and volume to reach acceptable cosmetic results, e.g., fingertip flap reconstruction with nail downsizing [31]. There are reports on tridimensional preoperative planning, a series of nerve allografts, or amputation followed by toe-to-hand transfer [32,33,34]. This diversity of methods shows that there is no universal treatment strategy. Ray amputation should not be considered a failure in management but discussed as an option to preserve the best functional outcome and limit the number of procedures. However, each case should be addressed individually. Ablation of the significantly overgrown and deformed thumb may seem like a dramatic choice, but immediate pollicization, transferring the index finger into the thumb position, can result in good cosmetic and functional results [35], as shown in our one-year-old patient (Figure 5). With experience in congenital hand surgery for thumb deficits, the early timing of the merger may allow for better adaptation to a new thumb. 

## 11. Nonsurgical Treatment

The pharmacological treatment of PROS has been constantly developing. The group of patients with isolated single-ray macrodactyly benefit from surgical treatment the most [19]. However, severe overgrowth conditions have been treated before with surgical, intravascular, and pharmacological protocols. Suzuki et al. reported the inhibition of PROS cells with low doses of rapamycin (sirolimus), an immunosuppressant used routinely after kidney transplantation, to lower the chance of rejection. Rapamycin binds with the cytotoxic protein FK-binding protein 12, and the complex then inhibits mTOR Complex 1. In the same study, the suppressed growth of normal control cells was also observed [36]. Thus, the potential use of rapamycin in the treatment of children affected with PROS should be carefully considered, taking numerous side effects into account. However, a recent review demonstrated only mild side effects in the treatment of isolated overgrowth with oral rapamycin [37]. Sandbank et al. reported good responses to topical sirolimus in the case of vascular malformations, such as in the KTS-part of the PROS spectrum [38]. The most recent application of alpelisib in the treatment of children with a severe presentation of the overgrowth spectrum is worth mentioning [17]. Alpelisib, a PIK3CA inhibitor previously well-known in oncology, has been primarily introduced in severe cases with general symptoms. However, good outcomes may result from expanding this therapy to children with isolated macrodactyly. In 2022, alpelisib was approved by the FDA for targeted therapy in children over two years of age. Current eligibility criteria include severe PROS cases requiring systemic treatment [39]. Morin et al. reported the successful treatment of two infants with life-threatening complications due to overgrowth. Half of the recommended minimal dosage was used, resulting in significant clinical improvement and no observed adverse effects [17]. We await the results of the ongoing multicenter study of Miransertib, yet another PIK3CA pathway inhibitor [40]. 

In summary, there are very limited data on the sufficient duration of treatment, including life-long treatment with the abovementioned drugs, with an emphasis on risks. Especially in young patients, pharmacological treatment strategies are limited to the most severe and life-threatening conditions. 

## 12. Long-Term Outcomes of Macrodactyly

**Figure 6:**
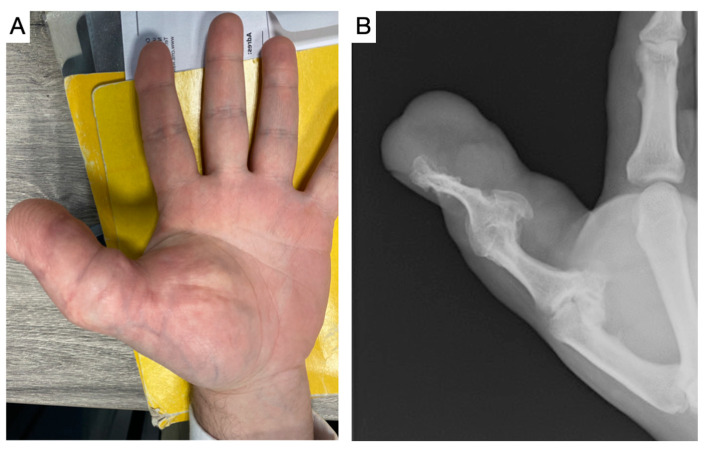
(**A**). Persistent growth in an isolated thumb macrodactyly in a 35-year-old patient after numerous soft tissue debulking and osteotomies. (**B**). Radiological picture with destruction of the interphalangeal and metacarpophalangeal joints.

The long-term observations of adults with macrodactyly present a challenging picture—most affected children and adults require at least two surgeries [3]. Persistent growth with secondary degenerative bone changes, despite surgical treatment, was observed in the adult population by Stor et al. [2]. Patients experience discomfort, poor cosmetic outcomes, and difficulties in basic functioning. In reality, patients and their caregivers may face a broad spectrum of psychological challenges at different stages of life. Psychotherapy, especially if offered at the beginning stages, plays a vital role in patients’ well-being. This may further help them transition to a new care team as they reach adulthood [29]. Even with extensive treatment during childhood, growth can continue later in life. The long-term follow-up of adult patients should be planned, such as in the case of a 35-year-old man who underwent numerous debulking and osteotomies, resulting in a dysfunctional thumb (Figure 6). Routine checkups may allow for earlier intervention and may help to preserve function; however, follow-up guidelines are yet to be established [2]. Moreover, some patients present with short-term complications, such as keloid formations when macrodactylous fingers undergo syndactyly release. Treatment may include methotrexate therapy [41]. 

## 13. Future Studies

Upcoming research may include noninvasive prognostic methods that estimate the rate and spectrum of overgrowth. Such a tool could greatly impact parents’ and surgeons’ decisions. To this day, no differences in the pathophysiology between static and progressive macrodactyly have been described. Current research on PIK3CA mutation analysis, including different pathogenic variants, has yet to prove its predictive value [42,43]. However, current studies have uncovered the role of osteogenesis and adipogenesis in macrodactyly, with further hopes of understanding the pathophysiology and treatment options [44,45]. With a confirmed PIK3CA mutation in a tissue biopsy, phenotyping may serve as a prognostic factor in PROS [13]. Furthermore, next-generation sequencing allows for the better identification of PIK3CA mutations, including mosaicism. This may serve as a future cornerstone for targeted therapy based on mutation variants [19]. Registries with significant inclusion rates may provide a better insight into the true picture of the population of children and adults with macrodactyly [6]. Such a database is fundamental for stable long-term follow-up and observation of both the natural history and effects of interventions, allowing us to become more aware of the actual impact that treatment may have. 

## 14. Conclusions

Macrodactyly is a rare congenital limb difference that can cause significant stigma and challenges for both the affected child and their caregivers. It presents as a spectrum of overgrowth that can be either an isolated mild congenital limb difference or the tip of the iceberg of a systemic entity. Differential diagnosis incorporates diagnostic measures to exclude underlying malignancy. The pathophysiology of macrodactyly and other overgrowth conditions is not yet fully understood. The broad spectrum of phenotypes requires a multidisciplinary team of consultants and a personalized approach to care. However, still advancing surgical treatment options and ongoing drug trials give hope for better patient outcomes. This is why it is worth presenting its complexity to a broad audience of pediatricians to raise awareness of conditions that may be first seen during routine child examinations. The initial conversation with parents can have a calming effect and can lead to further extensive specialist consultations to offer the best multidisciplinary treatment and support options. 

## Figures and Tables

**Table 1:** Individual system for the classification of macrodactyly [4].

Characteristic	Classification	
Growth	Static	Progressive
Associations	Isolated	Associated syndrome or anomalies
Structure	Lipomatous nerve territory orientated	Hyperostotic vascular malformation

**Table 2:** Examples of soft tissue tumors of the hand [21].

Soft Tissue Tumors of the Hand
Ganglion cyst
Tenosynovial Giant Cell Tumor
Lipoma
Schwannoma
Glomus Tumor
Vascular Tumors and Malformations
Superficial Fibromatoses
Synovial Chondromatosis
Soft Tissue Sarcomas, e.g., Fibrosarcoma, Rhabdomyosarcoma

**Table 3:** The most common PIK3CA-Related Overgrowth Spectrum (PROS) conditions [23].

Most Common PIK3CA-Related Overgrowth Spectrum (PROS) Conditions
KTS (Klippel–Trenaunay Syndrome)
CLOVES syndrome (Congenital Lipomatous Overgrowth, Vascular malformations, Epidermal nevi, Scoliosis/skeletal and spinal)
ILM (Isolated Lymphatic Malformation)
MCAP or M-CM (Megalencephaly-Capillary Malformation)
HME (HemiMegalEncephaly)/DMEG (Dysplastic MEGalencephaly)/Focal cortical dysplasia type II
HHML (HemiHyperplasia-Multiple Lipomatosis)
FIL (Facial Infiltrating Lipomatosis)
FAVA (FibroAdipose Vascular Anomaly)
Macrodactyly
Muscular HH (HemiHyperplasia)
FAO (FibroAdipose hyperplasia or Overgrowth)
CLAPO syndrome (Capillary malformation of the lower lip, Lymphatic malformation of the face and neck, Asymmetry of the face and limbs, and Partial or generalized Overgrowth)
Epidermal nevus, benign lichenoid keratosis, or seborrheic keratosis

**Table 4:** Overgrowth syndromes associated with an increased risk of neoplasms [24,25,26,27,28].

Generalized overgrowth
Beckwith–Wiedemann syndrome
Sotos syndrome
Simpson–Golabi–Behmel syndrome
Perlman syndrome
Weaver syndrome
Segmental overgrowth
PTEN hamartoma tumor syndrome
Bannayan–Riley–Ruvalcaba Syndrome
Macrocephaly cutis marmorata telangiectatica
Costello syndrome
Neurofibromatosis type 1
Gorlin syndrome
MEN 2B

## Data Availability

Data sharing is not applicable.
